# Regulation of FSH expression by differentially expressed miR-186-5p in rat anterior adenohypophyseal cells

**DOI:** 10.1371/journal.pone.0194300

**Published:** 2018-03-13

**Authors:** Dong-Xu Han, Yue Xiao, Chang-Jiang Wang, Hao Jiang, Yan Gao, Bao Yuan, Jia-Bao Zhang

**Affiliations:** Department of Laboratory Animals, College of Animal Sciences, Jilin University, Changchun, Jilin, P.R. China; Universitat des Saarlandes, GERMANY

## Abstract

Follicle-stimulating hormone (FSH) has key roles in animal reproduction, including spermatogenesis and ovarian maturation. Many factors influence FSH secretion. However, despite the broad functions of microRNAs (miRNAs) via the regulation of target genes, little is known about their roles in FSH secretion. Our previous results suggested that miR-186-5p targets the 3′ UTR of *FSHb*; therefore, we examined whether miR-186-5p could regulate FSH secretion in rat anterior adenohypophyseal cells. miR-186-5p was transfected into rat anterior pituitary cells. The expression of *FSHb* and the secretion of FSH were examined by RT-qPCR and ELISA. A miR-186-5p mimic decreased the expression of *FSHb* compared with expression in the control group and decreased FSH secretion. In contrast, both the mRNA levels and secretion of FSH increased in response to miR-186-5p inhibitors. Our results demonstrate that miR-186-5p regulates FSH secretion by directly targeting the *FSHb* 3′ UTR, providing additional functional evidence for the importance of miRNAs in the regulation of animal reproduction.

## Introduction

FSH, which is synthesized and secreted by the anterior pituitary gland, has important functions in both sexes in mammals. In males, FSH targets testis Sertoli cells to regulate spermatogenesis [1, 2]. In females, FSH stimulates ovarian follicle growth and maturation and promotes granulosa cell proliferation and differentiation [1, 3]. It is a heterodimeric glycoprotein composed of a common α subunit (α-GSU) and a unique β subunit (FSHb). FSH plays important roles in animal reproduction [4, 5] and is regulated by many factors. For example, multiples studies have shown that FSH is affected by the level of GnRH [6, 7]. In addition, Kiezun et al. showed that adiponectin plays a major role in FSH secretion during the estrous cycle [8]. However, little is known about the regulation of FSH secretion at the post-transcriptional level.

A microRNA (miRNA) is an ~22 nucleotide, short, non-coding RNA molecule that functions as a post-transcriptional regulator of gene expression by suppressing transcription or degrading the protein [9]. miRNAs regulate gene expression in animals, plants, and viruses [9]. There is increasing evidence for a role of miRNAs in hormone regulation. Hasuwa et al. indicated that miR-200b/miR-429 stimulates luteinizing hormone (LH) levels by targeting ZEB1 [10], and Nemoto et al. showed that miR-325-3p decreases LH secretion [11]. Furthermore, miR-26b is involved in growth hormone (GH) regulation [12] and miRNAs have been identified as regulators of gonadotropins [13, 14]. Zhang et al. indicated that miR-143 mediates the proliferative signaling pathway of FSH and further regulates estradiol production [15]. However, the regulatory role of miRNAs in FSH secretion is unclear.

In this study, to verify the interaction between *FSHb* mRNA and miR-186-5p, we mutated the target sites of miR-186-5p in the *FSHb* 3′ UTR. To determine whether miR-186-5p regulates FSH secretion, we measured the mRNA expression levels of *FSHb* and the secretion of the FSH hormone after the transfection of miR-186-5p into primary rat pituitary cells.

## Materials and methods

### Ethics statement

This study was carried out in strict accordance with the recommendations in the Guide for the Care and Use of Laboratory Animals of Jilin University. The rats were fed according to the cleanliness specifications. Animals were provided free access to food. Animal feed, litter, and drinking water were used after disinfection and sterilization, and the cage and litter were regularly cleaned and replaced. Housing and care procedures were in compliance with the provisions in the general guidelines of Animal Experimentation of Jilin University. Rats were euthanized by inhaling an anesthetic followed by carbon dioxide and pick the pituitary. Animal corpses were handled according to Harmless Treatment when the experiments were completed. The protocol was approved by the Institutional Animal Care and Use Committee of Jilin University (Permit Number: 20170605).

### Animals and cell culture

Ten 4-month-old and five 2-week-old healthy male Wistar rats were obtained from the School of Medical Science of Jilin University. Five male 4-month-old rats were used for cell culture and transfection experiments. Pituitaries were obtained from five 2-week-old rats, and seven tissues, including pituitary, heart, liver, spleen, lung, kidney, and brain tissues, were obtained from five 4-month-old rats. Pituitaries from rats of different ages were used to measure miRNA expression levels at various developmental stages. The seven tissues were used to measure the expression of miR-186-5p in different tissues. Rat primary cell culture was performed as described in a previous study [16].

### Flow cytometry analysis of rat anterior adenohypophysis cell apoptosis

Rat anterior adenohypophysis cell apoptosis was detected using the Annexin V-FITC/PI Apoptosis Kit following the manufacturer’s instructions (Multi Sciences, Hangzhou, China). After 24 h of transfection, cells were digested with trypsin without EDTA, centrifuged at 200 × *g* for 5 min, and 1–5 × 10^5^ cells were collected. Then, 5× Binding Buffer was diluted in double distilled water to obtain a 1× working fluid, and 500 μL was added to each tube. A blank control tube, Annexin V-FITC single-dye tube, PI single-dye tube, and sample tubes were established. Annexin V-FITC (5 μL) and PI (10 μL) were added to the two single-dye tubes. Sample tubes were supplemented with both 5 μL of Annexin V-FITC and 10 μL of PI. The apoptotic cells and dead cells were stained by PI and Annexin V-FITC and analyzed by flow cytometry in 2 h.

### Transfection and qRT-PCR

Rat pituitary cell transfection was performed according to the methods of our previous study [16]. Total RNA was extracted using the RNAprep Pure Cell/Bacteria Kit (Tiangen, Beijing, China) according to the manufacturer’s recommended protocol. RT-PCR and qRT-PCR were performed using the FastQuant RT Kit (with gDNase) and SuperReal PreMix Plus (SYBR Green) (Tiangen), respectively. The mRNA and miRNA primers are listed in Table 1.

**Table 1:** Primers used for RT-qPCR.

Primer name	Sequence (5′–3′)
U6 RT	CGCTTCACGAATTTGCGTGTCAT
miR-186-5p RT	CTCAACTGGTGTCGTGGAGTCGGCAATTCAGTTGAGAGCCCAAA
U6 F	GCTTCGGCAGCACATATACTAAAAT
U6 R	CGCTTCACGAATTTGCGTGTCAT
miR-186-5p F	ACACTCCAGCTGGGCAAAGAATTCTCCTTT
universal reverse	CTCAAGTGTCGTGGAGTCGGCAA
GAPDH F	GGAAACCCATCACCATCTTC
GAPDH R	GTGGTTCACACCCATCACAA
FSHb F	ATACCACTTGGTGTGAGGGC
FSHb R	TAGAGGGAGTCTGAGTGGCG

### Construction of the reporter plasmids

According to the genome sequence information for the rat *FSHb* 3′ UTR (GenBank Accession No. NM_001007597.2), PCR primers were designed to amplify the full length of the *FSHb* 3′ UTR. The forward primer was GCGCTCGAGGGAACAATGGACATTGCC and the reverse primer was AATGCGGCCGCTTCATCAGTAGCACTTTTA. The amplified fragment was cloned between the XhoI and NotI sites of the pmiR-RB-REPORT^TM^ plasmid to construct the pmiR-FSHb-3′UTR-WT plasmid. Two target sites of miR-186-5p and the *FSHb* 3′ UTR were mutated, i.e., TTCTTTA to AAGAAAT and ATTCTTT to TAAGAAA, to obtain pmiR-FSHb-3′UTR-MUT (S1 File). Reporter plasmid construction and the confirmation of construct products were performed by Guangzhou Ribobio Biotech Co., Ltd. (Guangzhou, China).

### FSH detection

After transfection for 24 h, 50 μL of the cell supernatant was collected to detect FSH levels using the Rat FSH ELISA Kit (Haling Biotech Co., Ltd., Shanghai, China).

### Statistical analysis

Data are presented as the means ± SD from three independent experiments. Data were analyzed using SPSS 19.0. One-way ANOVA was used to determine significant differences; p < 0.05 was considered significant.

## Results

### MiR-186-5p targets the 3′ UTR of *FSHb* mRNA

**Fig 1:**
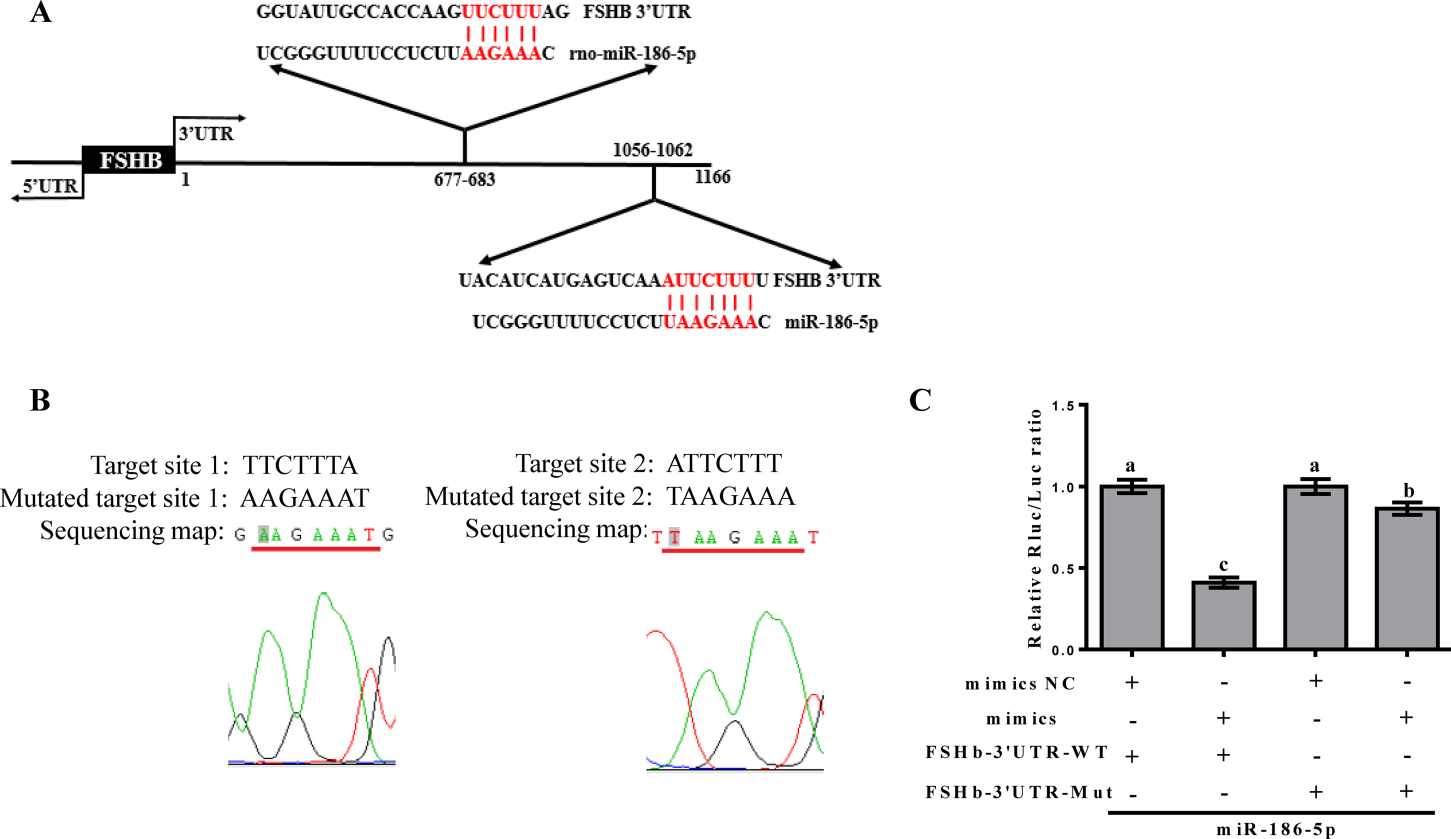
MiR-186-5p targets the 3′ UTR of *FSHb* mRNA. (A) The seed match regions predicted by TargetScan for the *FSHb* 3′ UTR and miR-186-5p are shown in red. (B) Mutations in the target sites for miR-186-5p. The sequence was identified using a sequencing map. (C) After 48-h of the co-transfection of pmiR-FSHb-3′UTR-WT and pmiR-FSHb-3′UTR-MUT with the miR-186-5p negative control and mimic, the relative luciferase activity was measured. The normalized luciferase activity for the controls was set to 1. All experiments were repeated at least three times. Data are shown as means ± SD. Statistical significance was analyzed by one-way ANOVA, p < 0.05 was considered significant and differences are marked with different letters.

In our previous study, a luciferase reporter assay indicated that miR-186-5p mimics reduce luciferase activity by 32% compared with that of a negative control group [16]. To further verify the interaction between miR-186-5p and *FSHb*, we predicted the target site of miR-186-5p in the *FSHb* 3′ UTR using TargetScan (Fig 1A), and mutated the target site TTCTTTA to AAGAAAT and ATTCTTT to TAAGAAA (Fig 1B). Next, we constructed pmiR-FSHb-3′UTR-MUT and co-transfected miR-186-5p mimics and pmiR-FSHb-3′UTR-MUT into 293T cells. Co-transfection of miR-186-5p mimics and pmiR-FSHb-3′UTR-WT into 293T cells led to a greater than 40% reduction in luciferase activity; moreover, when the miR-186-5p binding site of the *FSHb* 3′ UTR was replaced with a mutant sequence (pmiR-FSHb-3′UTR-MUT), there was an increase in luciferase activity compared with that of the control group (Fig 1C). Therefore, miR-186-5p and *FSHb* had a direct interaction, indicating that miR-186-5p could regulate FSHb expression.

### Detection of differential miR-186-5p expression at various developmental stages in the pituitary and in other rat tissues

**Fig 2:**
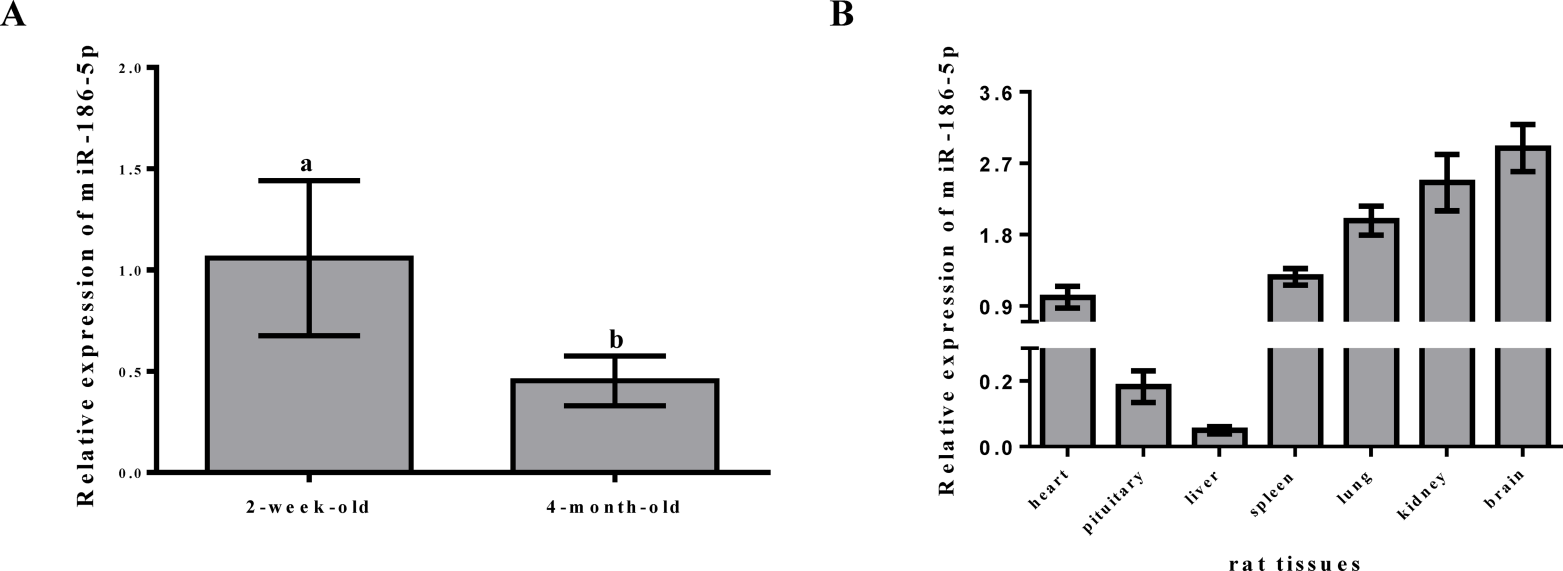
Detection of the differential expression of miR-186-5p at various developmental stages of the pituitary and in various tissues in rats. (A) The relative expression level of miR-186-5p was detected in 2-week-old and 4-month-old rats, and U6 was used as an internal standard for qRT-PCR. (B) The relative expression of miR-186-5p was detected by qRT-PCR with U6 as an internal control in seven tissue types from 4-month-old rats, i.e., the pituitary, heart, liver, spleen, lung, kidney, and brain. All experiments were repeated at least three times. Data are shown as means ± SD. Statistical significance was analyzed by one-way ANOVA, p < 0.05 was considered significant, and differences are marked with different letters.

We detected miR-186-5p expression in 2-week- and 4-month-old rats to examine expression differences among developmental stages in the rat pituitary. We detected miR-186-5p expression in both stages, and we found that compared with levels in the non-sexual maturity period, miR-186-5p was down-regulated in the sexual maturity period (Fig 2A). We examined the expression of miR-186-5p in seven tissues of 4-month-old rats, including the pituitary, heart, liver, spleen, lung, kidney, and brain. miR-186-5p was expressed in all of these tissue types, and was particularly highly expressed in the lung, kidney, and brain (Fig 2B).

### Effect of miR-186-5p transfection into rat primary pituitary cells

**Fig 3:**
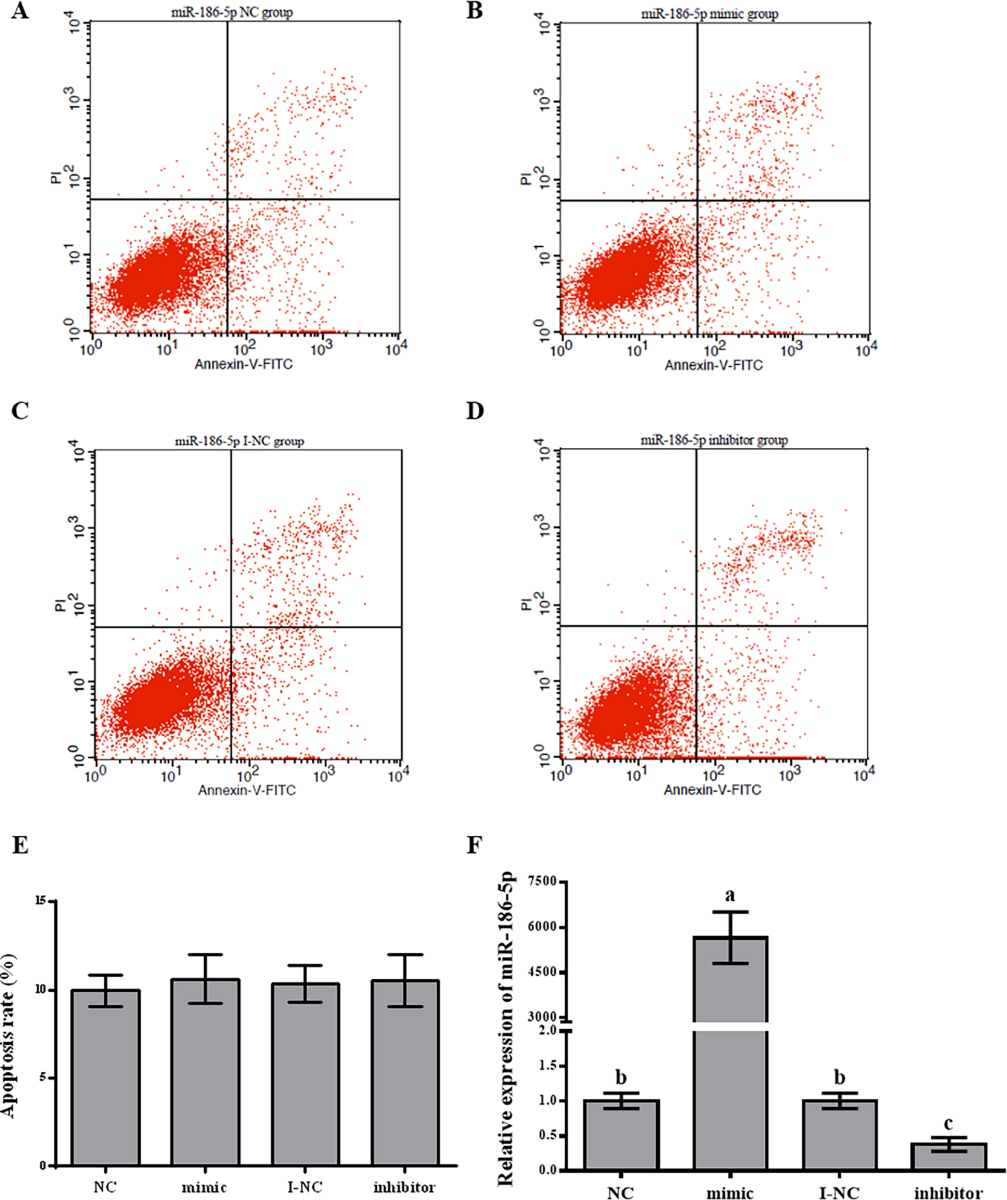
Effect of miR-186-5p transfection into rat primary pituitary cells. After transfection for 24 h, rat anterior adenohypophyseal cells were stained with Annexin V-FITC/PI to observe apoptosis by flow cytometry. (A) miR-186-5p mimic negative control group (NC). (B) miR-186-5p mimic group. (C) miR-186-5p inhibitor negative control group (I-NC). (D) miR-186-5p inhibitor group. (E) The apoptosis rate of anterior pituitary cells after transfection with miR-186-5p mimic NC, mimic, I-NC and inhibitor. (F) The relative expression of miR-186-5p with miR-186-5p mimics/inhibitor transfection. All experiments were repeated at least three times. Data are shown as means ± SD. Statistical significance was analyzed by one-way ANOVA, p < 0.05 was considered significant, and differences are marked with different letters.

For quality control, we used flow cytometry to detect rat anterior pituitary cell apoptosis after transfection with 100 nM miR-186-5p negative control, inhibitor negative control, mimic, and inhibitor for 24 h. We did not detect significant differences among the negative control groups and transfection groups (Fig 3A–3D). We then examined the expression of miR-186-5p after transfecting cells with 100 nM miR-186-5p negative control, inhibitor negative control, mimic, and inhibitor. The overexpression of miR-186-5p significantly increased the expression of miR-186-5p (5653.605-fold, p < 0.01) compared with expression in the negative control group (Fig 3E). When we inhibited the expression of miR-186-5p, the level of miR-186-5p decreased (0.382-fold, p < 0.01) compared with levels in the inhibitor negative control group (Fig 3F). Accordingly, the over-expression or inhibition of miR-186-5p was successful.

### *FSHb* expression and FSH secretion after the overexpression or inhibition of miR-186-5p

**Fig 4:**
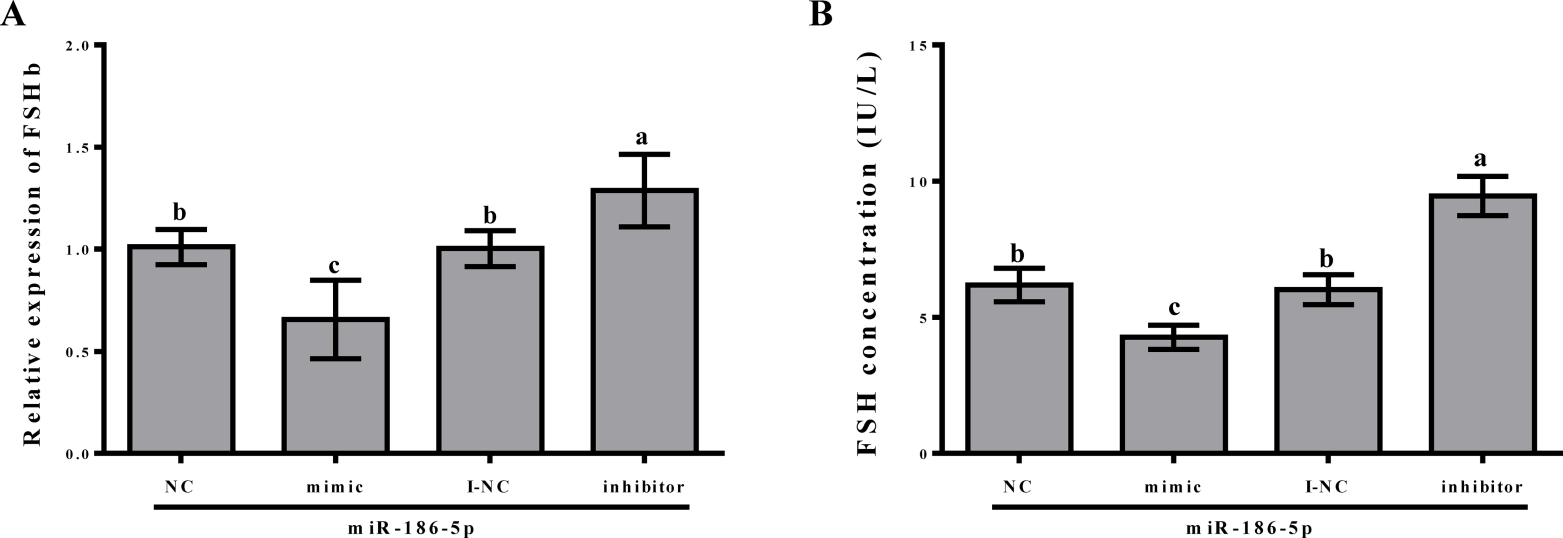
mRNA expression levels of *FSHb* and the level of FSH secretion after the overexpression or inhibition of miR-186-5p. (A) The relative expression of *FSHb* in miR-186 NC, mimic, I-NC, and inhibitor groups. (B) ELISA was used to examine the supernatant from pituitary cells after transfection for 24 h with miR-186 NC, mimic, I-NC, and inhibitor. All experiments were repeated at least three times. Data are presented as means ± SD. Statistical significance was analyzed by one-way ANOVA, p < 0.05 was considered significant, and differences are marked with different letters.

We further verified the interaction between miR-186-5p and *FSHb* in rat anterior pituitary cells and examined the role of this interaction in the regulation of animal reproduction. Specifically, rat primary pituitary cells were transfected using 100 nM negative control (NC), miR-186-5p mimic, inhibitor negative control (I-NC), and miR-186-5p inhibitor; after 24 h, the level of *FSHb* was examined by quantitative RT-PCR. *FSHb* mRNA levels decreased significantly (0.66-fold, p < 0.05; Fig 4A) after the overexpression of miR-186-5p compared with the levels in the NC group. *FSHb* expression levels were significantly higher in the miR-186-5p inhibitor group than in the I-NC group (1.29-fold, p < 0.05; Fig 4A). Since the over-expression and inhibition of miR-186-5p led to a significant reduction and increase in *FSHb* levels, respectively, we examined whether the same trends were observed for FSH secretion by ELISA after transfection for 24 h. FSH secretion and *FSHb* expression exhibited similar patterns in the four groups. The overexpression of miR-186-5p significantly decreased FSH secretion (6.19 ± 0.62 IU/L vs. 4.27 ± 0.45 IU/L, p < 0.05; Fig 4B). The inhibition of mir-186-5p significantly increased FSH secretion (6.02 ± 0.55 IU/L vs. 9.45 ± 0.72 IU/L, p < 0.05; Fig 4B).

These results demonstrated that miR-186-5p can regulate FSHb by inhibiting the expression of *FSHb* and by decreasing the secretion of FSH via direct binding to the *FSHb* 3′ UTR.

## Discussion

Analyzing changes in the relative luciferase activity of a reporter gene after co-transfection of a candidate miRNA with a mutant reporter gene vector is an important strategy to understand interactions between miRNAs and target genes. In some cases, mutations in miRNAs and target sites in genes do not affect luciferase activity compared with co-transfection with a reporter construct containing a wild-type 3′ UTR for the target gene [16–18]. However, in our study, when we co-transfected miR-186-5p mimics and pmiR-FSHb-3′UTR-WT into 293T cells, the luciferase activity was reduced by more than 40%; after mutating two target sites, luciferase activity was slightly higher, but still significantly different from wild-type levels. Interestingly, in 2014, Fu et al. reported that let-7a, miR-9, and miR-129-5p each had two target sites in *FOXP2*, and three mutant luciferase reporter constructs with mutations in one or two target sites were made. When only one binding site was mutated, luciferase activity was repressed by miRNAs, while mutations in both target sites led to the abolishment of regulatory effects [19]. Our results indicated that there are additional target sites that were not predicted using TargetScan. However, the increase in luciferase activity indicated that the predicted target sites were correct and miR-186-5p and *FSHb* had a direct interaction.

miRNAs act as regulators in many biological processes, including developmental timing, cell death, cell proliferation, and hematopoiesis [20]. miRNAs function in a tissue-specific and time-dependent manner [21]. They have effects in the pituitary, a critical endocrine organ. In pituitary adenomas, increasing studies have provided evidence for the roles of miRNAs in various cellular processes; they have pathobiological significance [22]. For example, miR-145 and miR-15a are down-regulated in GH and pituitary adenomas, respectively [23, 24]. Gentilin et al. found that miR-26a could directly target PRKCD and is involved in cell cycle regulation in ACTH-secreting pituitary adenomas [25]. They indicated that miRNA-mediated regulation has complex cellular outcomes and miRNAs can also mediate the regulation of cell cycle, cell growth, and apoptosis pathways. Furthermore, miRNAs also have a regulatory role in the normal pituitary. For instance, miR-26b is involved in the development of the pituitary in mice [12]. In 2013, Zhang et al. found that miR-7 might function in the pituitary in pigs, thereby influencing body growth [26]. miRNAs can also regulate hormone secretion. For example, miR-325-3p is associated with the immobilization-induced suppression of LH secretion [11], and miR-375 is involved in ATCH secretion [27]. In our previous study, miR-186-5p expression was significantly differentially expressed (0.45-fold difference) between the non-sexual maturity developmental stage and the sexual maturity developmental stage in the rat pituitary [16]. Accordingly, miR-186-5p is a potential regulator of pituitary development and reproduction.

Most miRNAs are expressed in a highly tissue-specific manner in the late stages of division, but not in early development [21]. According to previous studies, miR-1 is highly expressed in the heart, but not in the brain or liver in human adults and mice [28, 29]. In 2014, miR-1, miR-133a, miR-133b, and miR-206 were validated as muscle-specific miRNAs in rat [30]. In 2015, Alessandro et al. identified 10 novel and 4 known heart-specific miRNAs in the left ventricle in sheep [31]. Moreover, in our study, miR-186-5p exhibited different degrees of expression in the rat heart, pituitary, liver, spleen, lung, kidney, and brain. Its expression tended to be high in the kidney and brain, but low in the pituitary and liver. Although miR-186-5p was not a tissue-specific miRNA in these rat tissues, its differential expression suggests that it is a candidate for further research.

The miRNA miR-186-5p is an important tumor suppressor and has been observed in many types of human cancers, including gastric cancer, hepatocellular cancer, and glioblastoma multiforme [32–34]. In 2016, Huang et al. indicated that miR-186 suppresses cell proliferation and metastasis by targeting MAP3K2 in non-small cell lung cancer [35]. In addition, in nonmelanoma skin cancer, miR-186-5p could be a novel noninvasive biomarker for detection [36]. Thananya et al. showed that miR-186-5p might be a specific hepatic miRNA that responds to hepatitis B virus and predicts the response to pegylated-interferon alpha-2a [37]. When E6/E7 is silenced, miR-186-5p is down-regulated in HPV18-positive HeLa cells [38]. These studies demonstrated that miR-186-5p acts as an anti-oncogenic marker and is involved in the control of cell proliferation, senescence, and apoptosis. Despite a number of studies indicating that miR-186-5p is associated with human cancers, its role in hormone secretion is unclear. Our results showed that the overexpression of miR-186-5p down-regulates *FSHb* expression and decreases FSH secretion in rat anterior pituitary cells, providing evidence that hormone secretion in the pituitary is regulated by miRNAs.

FSH, as a pituitary hormone, is critical for the regulation of reproduction in mammals [39]. Therefore, it is essential to determine the mechanism and timing of FSH regulation. Many factors determine *FSHb* expression and FSH secretion, including hormones, follistatin, and single nucleotide polymorphisms [40–42]. A novel single nucleotide polymorphism influences the FSH beta-subunit and the quality and fertility of bull semen [43]. Additionally, a cis-regulatory element influences FSHb expression levels in bovine cells [44]. FSH secretion is strongly associated with GnRH, bio-available activin, and steroid hormones [45–47]. However, few studies have reported a role of miRNAs in the regulation of FSH secretion. In 2015, Lannes et al. reported that the miR-132/212 pathway is associated with the stimulation of FSH secretion by GnRH [48]. Ye et al. identified 10 up-regulated and 3 down-regulated miRNAs that are likely to directly target the porcine *FSHb* 3′ UTR after GnRH treatment [49]. In 2016, miR-361-3p was found to regulate FSH by directly interacting with *FSHb* in porcine anterior pituitary cells [4]. Furthermore, we previously found that miR-21-3p and miR-433 both decrease FSH secretion by regulating FSHb [16]. In this study, the overexpression of miR-186-5p inhibited FSH secretion. Our results provide additional evidence for the regulatory role of miRNAs in FSH secretion.

Taken together, our results showed that miR-186-5p down-regulates the expression of *FSHb* and inhibits the secretion of FSH. These findings provide additional support for the regulatory functions of miRNAs in reproduction.

## Supporting information

S1 File Construction of the pmiR-FSHb-3′UTR-MUT reporter plasmid. (PDF)

S1 Table Raw data for Figs 1–4. (XLSX)
