# Supplementary material for: Regulation of FSH expression by differentially expressed miR-186-5p in rat anterior adenohypophyseal cells
Source: PLoS One. 2018 Mar 13;13(3):e0194300. doi: 10.1371/journal.pone.0194300 (PMC5849326; doi:10.1371/journal.pone.0194300)
Supplement: S1 File — (PDF) [file pone.0194300.s001.pdf]

S1 File. Construction of pmiR-FSHb-3’UTR-MUT reporter plasmid

The full-length 3’UTR of rat FSHb mRNA was cloned between the XhoI and NotI sites of the pmiR-RB-REPORT^{TM} plasmid. To disrupt the binding site of the FSHb 3’UTR, the target sequence TTCTTTA (707-713) was mutated into AAGAAAT, and ATTCTTT (1093-1099) was mutated into TAAGAAA, forming the pmiR-FSHb-3’UTR-MUT plasmid. The primers used in the colony experiment were as follows:

FSHb F: GCGCTCGAGGGAACAATGGACATTGCC
FSHb R: AATGCGGCCGCTTCATCAGTACGACTTTA
FSHb-MUT F: CCACCAAGAAGAAATGTTAAAGGAAAAGAAAAA
FSHb-MUT R: CCTTTAACATTTCTTCTTGGTGGCAATACCTTG
FSHb-MUT1 F: TGAGTCAATAAGAAATAAAGCTGTTAACTCTTT
FSHb-MUT1 R: CAGCTTTATTTCTTATTGACTCATGATGTATTT

The PCR colony was identified after purification of the PCR product, enzyme cleavage, purification of the cleavage product, connection and convention.

The plasmid was extracted from the colony, and the sequence was identified by a sequencing company. The results of sequencing (Fig 1A and B) showed that the target sequences were mutated successfully.

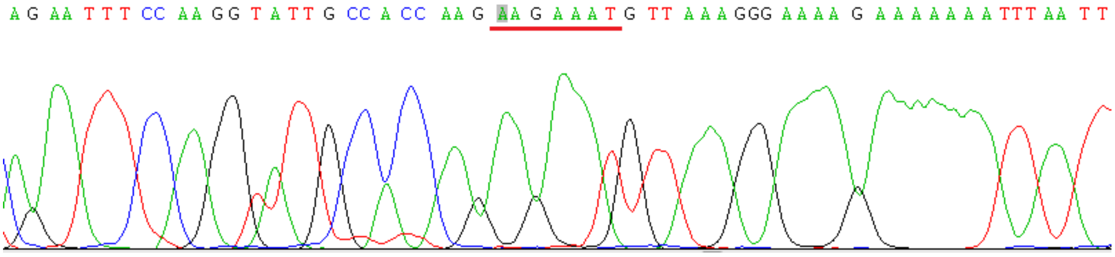

Fshb-MUT1

A

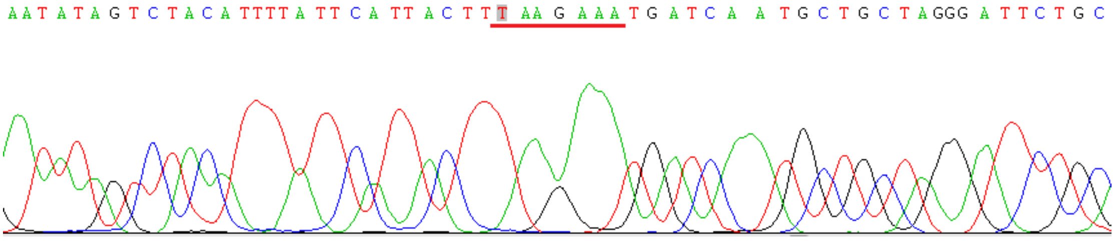

Fshb-MUT2

B

S1. Fig 1. DNA sequence peak map. (A) Sequence of the extracted plasmid; the target sequence was mutated into AAGAAAT. (B) Sequence of the extracted plasmid; the target sequence was mutated into TAAGAAA.
